# Late complications after Cabrol technique in a case with Marfan syndrome

**DOI:** 10.1002/ccr3.984

**Published:** 2017-05-16

**Authors:** Shin‐ichi Ohki, Ippei Takazawa, Hirohiko Akutsu, Yoshio Misawa

**Affiliations:** ^1^ Division of Cardiovascular SurgeryJichi Medical University3311‐1 YakushijiShimotsukeTochigi329‐0498Japan

**Keywords:** Annuloaortic ectasia, aortic root replacement, Cabrol technique, coronary artery aneurysm, Marfan syndrome

## Abstract

A patient with Marfan syndrome underwent aortic root replacement with the Cabrol technique at 37 years of age. She underwent a surgical repair for an aneurysm in the right coronary at 58 years of age, followed by a surgical repair for a pseudoaneurysm of the left coronary artery at 64 years of age.

## Introduction

Aortic root replacement surgery is well known to provide excellent clinical results. However, long‐term complications related to operative techniques and/or newly developing aortic diseases might require additional surgical interventions. Herein, we report a patient with Marfan syndrome who required repeated surgery for complications related to the Cabrol technique.

## Case Report

**Figure 1:**
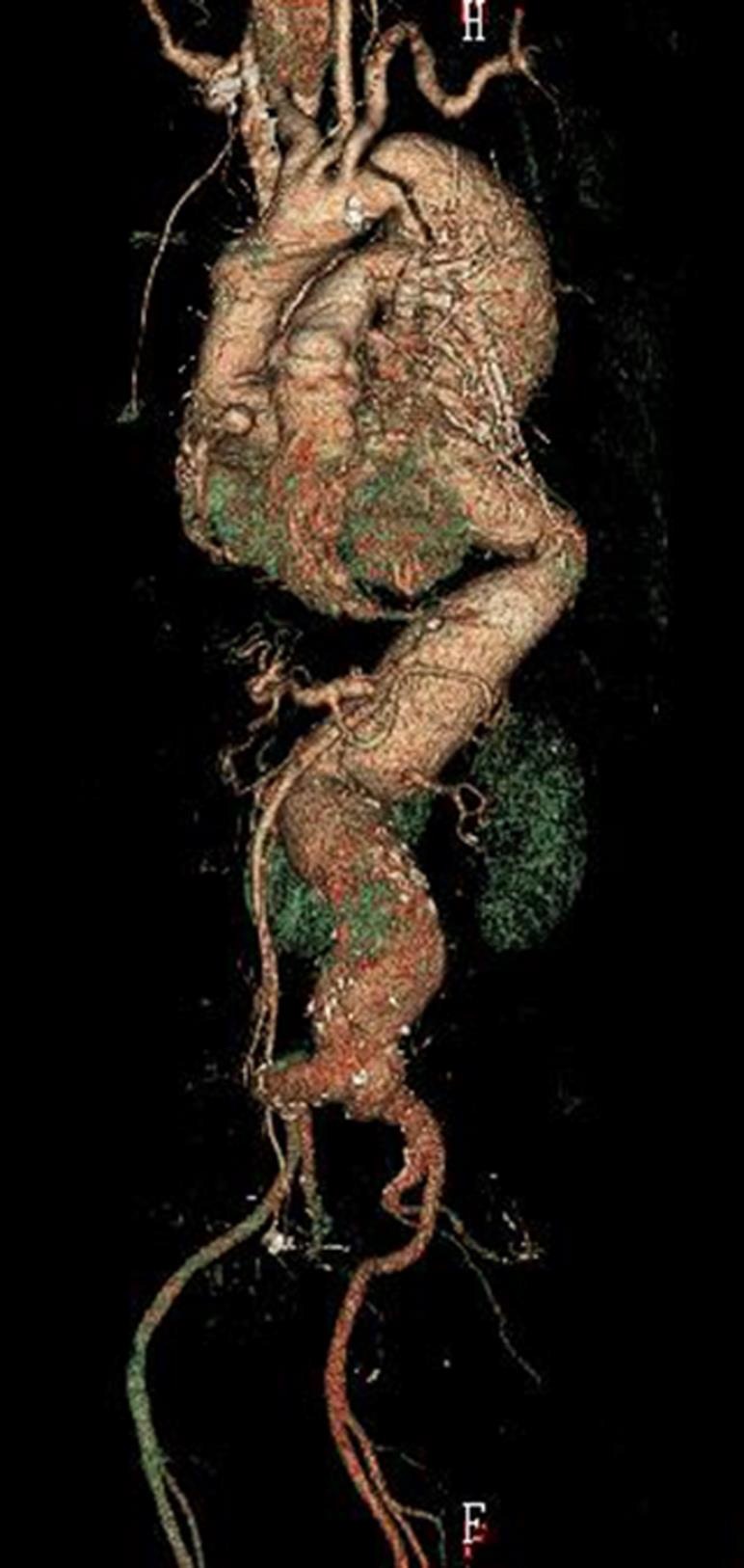
Follow‐up CT 18 years after the initial surgery. The descending and abdominal aortae with a pseudolumen are enlarged.

**Figure 2:**
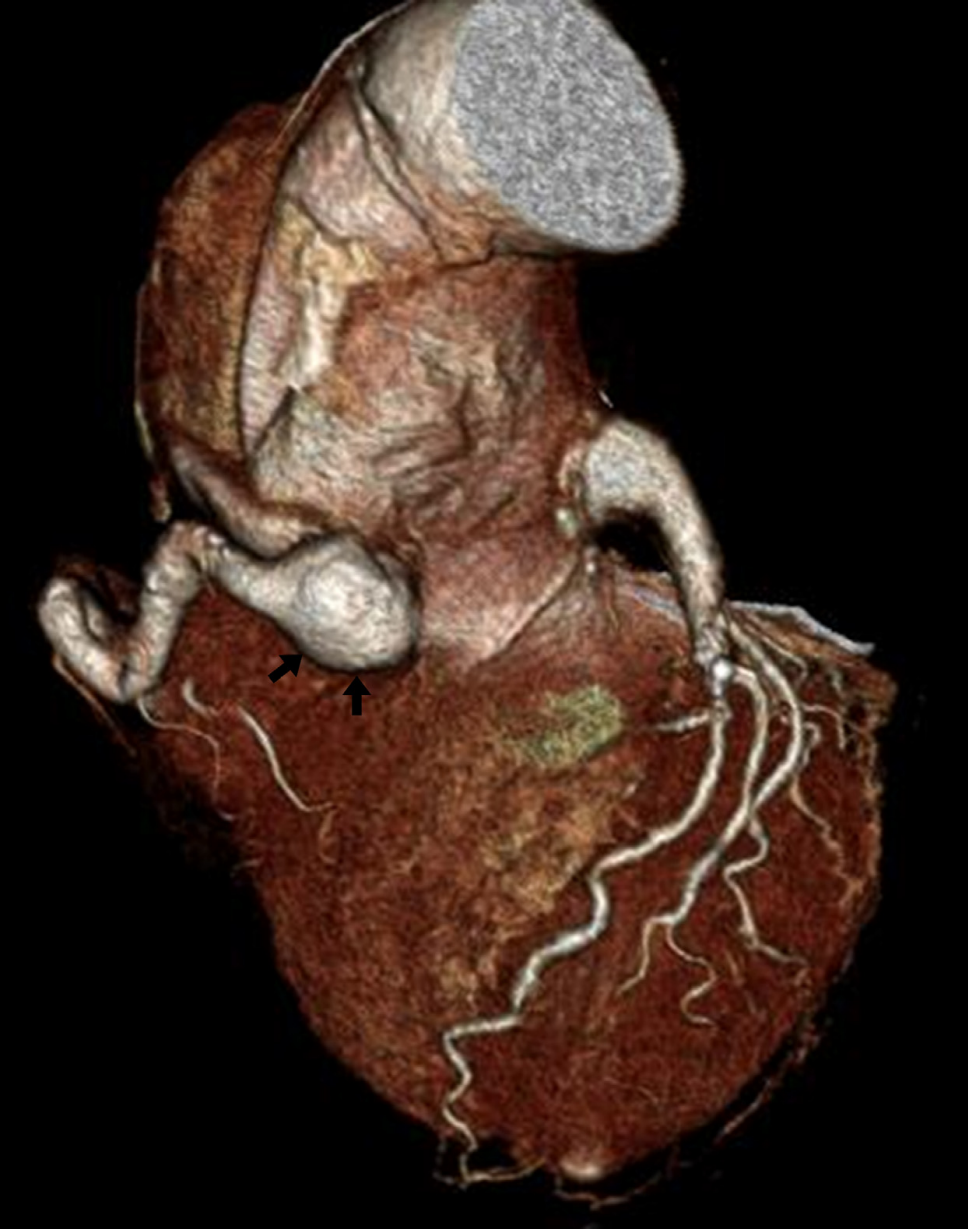
Chest CT before the third operation. The anastomotic site of the right coronary artery after the Cabrol technique is markedly enlarged (black arrows), while that of the left coronary artery is slightly enlarged.

**Figure 3:**
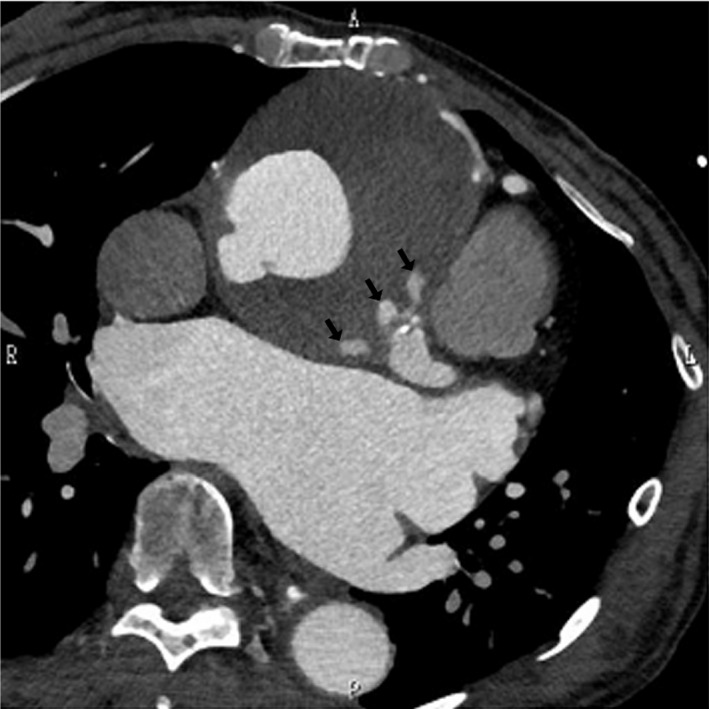
Chest CT before the fourth operation. It showed a pseudoaneurysm with leakage (black arrows) from the anastomotic site of the left coronary artery.

A 64‐year‐old woman with Marfan syndrome presented with heart failure. When she was 37 years of age, she had undergone aortic root replacement with the Cabrol technique, whereby a composite graft of the ascending aorta and aortic valve was implanted and a prosthetic vascular graft of 10 mm in diameter was interposed between the right and left coronary arteries. For the monitoring of possible development of cardiovascular diseases, serial follow‐up studies with chest computed tomography (CT) were scheduled, which showed dilatation of the descending aorta and abdominal aorta (Fig. 1). She thus underwent replacement of the descending and abdominal aortas at the age of 56 years. Thereafter, enlargement of the initial (Cabrol technique) anastomotic site in the right coronary artery progressed, associated with an aortic arch dissecting aneurysm (Fig. 2). At the age of 58 years, she underwent aortic arch replacement and resection of the enlarged anastomotic site of the right coronary artery, followed by reconstruction of the right coronary artery. A stenotic lesion of the left anterior descending artery required concomitant coronary artery bypass grafting. Additional work‐up studies revealed progressive mitral valve regurgitation and a pseudoaneurysm caused by anastomotic leakage from the anastomotic site of the left coronary artery (Fig. 3). She underwent mitral valve replacement surgery involving a mechanical valve and repair of the pseudoaneurysm. Severe adhesion around the anastomotic sites prevented resection of the residual dilated aortic wall around the coronary ostium. Thus, we chose palliative repair of the aneurysm. The postoperative course was uneventful (Fig. 4).

## Discussion

The Cabrol technique provides excellent clinical results for aortic root replacement when treating annuloaortic ectasia 1, although some authors have reported that long‐term survival after the Cabrol technique is shorter than that after the Carrel patch technique. Anastomotic complications related to the Cabrol technique have also been reported 2, 3, 4, 5, 6. Aneurysmal changes with or without anastomotic leakage are serious complications that can occur long after the surgery has taken place. The Cabrol and original Bentall techniques leave the aortic wall around the coronary orifices. When a patient has aortopathy, such as cystic medial necrosis in Marfan syndrome, an aneurysmal change might occur at the anastomotic site. Kazui and colleagues reported that pseudoaneurysms of the coronary artery or distal aorta occurred in the original Bentall and Cabrol procedures 6. Thus, we abandoned these techniques 20 years ago, preferring to use a small Carrel patch technique (button technique) to minimize the residual aortic wall around the coronary orifices. We use the aortic wall as a margin for the seam of the anastomosis. Mok and colleagues reported excellent long‐term clinical results after the button technique 7.

Ideally, the residual aortic wall at the reoperation after the Cabrol procedure should be resected; however, postoperative adhesion often prevents the surgeon from doing so. Repair of a pseudoaneurysm is also employed for this treatment 4. In our case, resection of the residual dilated aortic wall around the right coronary artery orifice was possible, but the left coronary artery could not be clearly dissected; we therefore chose surgical repair of the pseudoaneurysm.

Stenotic complications after the Cabrol technique are also reparable. Maekawa and colleagues reported a case of pannus resection and coronary artery reconstruction by the Piehler procedure 8. Tsai and colleagues salvaged the occluded left Cabrol limb 3 months after the Cabrol operation by coronary artery bypass grafting 9.

In conclusion, anastomotic complications in the coronary artery occurred many years after the Cabrol procedure. Careful follow‐up studies are essential to diagnose such complications without delay, particularly in patients with Marfan syndrome.

## Authorship

SO, IT, and HA: participated in the design of this study and helped in competing an initial manuscript. YM: edited the final version of the manuscript. All authors read and approved the manuscript.

## Conflict of Interest

None declared.

## References

[1] Aoyagi, S. , K. Kosuge , H. Akashi , A. Oryoji , and K. Oishi . 1994 Aortic root replacement with a composite graft: results of 69 operations in 66 patients. Ann. Thorac. Surg. 58:1469–1475. 7979677 10.1016/0003-4975(94)91937-2

[2] Bachet, J. , J. L. Termignon , B. Goudot , G. Dreyfus , A. Piquois , D. Brodaty , et al. 1996 Aortic root replacement with a composite graft, factors influencing immediate and long‐term results. Eur. J. Cardiothorac. Surg. 10:207–213. 8664022 10.1016/s1010-7940(96)80298-3

[3] Tatsumi, Y. , H. Ohashi , A. Murakami , Y. Kawase , H. Furuta , and M. Ohnaka . 1996 Surgical results of total replacement of aortic root. Kyobu Geka 49:209–213. 8709427

[4] Yamashita, C. , K. Ataka , M. Yoshida , Y. Tsuji , T. Yamashita , K. Nakagiri , et al. 1998 Surgical results of composite graft replacement of the aortic root aneurysm. Ann. Thorac. Cardiovasc. Surg. 4:78–82. 9577002

[5] Sugawara, Y. , T. Shimakura , S. Kihara , S. Tanaka , N. Saitoh , and M. Imamaki . 1998 A combination of reoperation for pseudoaneurysm following the Cabrol procedure and total aortic arch replacement in a patient with Marfan syndrome—a case with an aberrant right subclavian artery. Jpn J. Thorac. Cardiovasc. Surg. 46:1041–1046. 9847586 10.1007/BF03217871

[6] Kazui, T. , K. Yamashita , H. Terada , N. Washiyama , T. Suzuki , K. Ohkura , et al. 2003 Late reoperation for proximal aortic and arch complications after previous composite graft replacement in Marfan patients. Ann. Thorac. Surg. 76:1203–1207. 14530012 10.1016/s0003-4975(03)00719-7

[7] Mok, S. C. , W. G. Ma , A. Mansour , P. Charilaou , S. Peterss , M. Tranquilli , et al. 2016 Twenty‐five year outcomes following composite graft aortic root replacement. J. Card. Surg. 32:99–109. 27966257 10.1111/jocs.12875

[8] Maekawa, Y. , Y. Yoshimura , T. Uchida , C. Kim , R. Miyazaki , E. Ohba , et al. 2012 Re‐do operation for coronary stenosis after Cabrol procedure: report of a case. Kyobu Geka 65:579–582. 22750836

[9] Tsai, C. L. , H. J. Wei , S. R. Hsieh , and Y. Chang . 2004 Salvage of left Cabrol limb occlusion by minimally invasive direct coronary artery bypass grafting. Interact. Cardiovasc. Thorac. Surg. 3:678–681. 17670339 10.1016/j.icvts.2004.08.005

